# The E3 Ligase RNF8 Promotes Ubiquitination and Degradation of ChREBPα During Liver Stress Response

**DOI:** 10.1096/fj.202600106R

**Published:** 2026-04-29

**Authors:** Yuee Zhao, Sujuan Wang, Jian Zhang, Sarah Cooke, Gary Zhang, Zifeng Zhao, Joon Oh, Xin Tong, Lei Yin

**Affiliations:** ^1^ Department of Molecular & Integrative Physiology University of Michigan Medical School Ann Arbor Michigan USA; ^2^ Caswell Diabetes Institute University of Michigan Medical School Ann Arbor Michigan USA; ^3^ Department of Nephrology The First Affiliated Hospital of Kuming Medical University Yunnan Province P. R. China; ^4^ Department of Infectious Diseases, the Second Xiangya Hospital Central South University Changsha Hunan Province P. R. China; ^5^ Department of Infectious Diseases, Xiangya Hospital Central South University Changsha Hunan Province P. R. China; ^6^ Neurosciences Graduate Program Case Western Reserve University School of Medicine Cleveland Ohio USA; ^7^ Shanxi Huanghe Institute of Traditional Chinese Medicine Taiyuan City Shanxi Province China

## Abstract

As the most common chronic liver disease, MASLD can progress to metabolic dysfunction‐associated steatohepatitis (MASH) driven by accumulated metabolic and inflammatory stresses. We previously reported that liver ChREBPα protein is markedly downregulated in mouse models of diet‐induced MASH and hepatotoxin‐induced liver injury. Yet the impact of stress pathways on hepatocyte ChREBPα proteolysis has not been examined. Here, we show that a combined metabolic (palmitate, PA) and inflammatory (TNFα) stress signal promotes ubiquitination and proteasome‐mediated degradation of ChREBPα in hepatocytes. More importantly, we identify the stress‐induced E3 ligase RNF8 as interacting with and promoting ChREBPα ubiquitination and degradation in a JNK2‐dependent manner. In vivo, acute depletion of JNK2 or RNF8 stabilizes ChREBPα in mouse liver, increases some but not all ChREBPα transcriptional targets, and reduces diet‐induced liver steatosis, inflammation, and fibrosis. Overall, our findings reveal the biochemical machinery underlying stress‐induced ChREBPα proteolysis and suggest that targeting RNF8‐mediated ChREBPα ubiquitination could be a new strategy for treating MASH.

## Graphical abstract

During MASH development, increasing hepatic stress signals, particularly a combination of lipotoxicity (palmitate) and inflammation (TNFα), trigger JNK, the key stress‐activated protein kinase. Activated JNK recruits RNF8, a stress‐induced E3 ligase, to promote ubiquitination and proteasomal degradation of the ChREBPα protein.

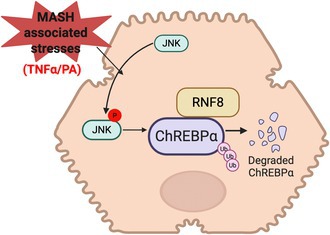

## 1 Introduction

As the most common chronic liver disease, metabolic dysfunction‐associated steatotic liver disease (MASLD) affects up to 25% of the global population [1]. While most MASLD patients have no significant symptoms, about 10%–15% can progress to its more severe form, namely metabolic dysfunction‐associated steatohepatitis (MASH), which can eventually lead to liver cirrhosis, liver failure, or liver cancer. MASH is characterized by lobular inflammation, hepatocyte ballooning, and excessive extracellular matrix accumulation [2, 3, 4]. It is widely accepted that MASH represents a state of chronic stress in the liver [5], resulting from a combination of mitochondrial dysfunction [6, 7], ER stress [8], oxidative stress [9], and impaired proteostasis [10]. However, the relative contribution and timing of specific stress pathways in MASH progression remain unclear. Stress‐related signaling pathways have been implicated in the pathogenesis of MASLD/MASH, especially the c‐Jun N‐terminal kinase (JNK) pathway [5, 11, 12], which functions as a stress‐activated protein kinase (SAPK) in response to various internal and external stressors to regulate multiple cellular responses.

The bHLH/ZIP transcription factor ChREBP⍺ is highly expressed in hepatocytes and activates lipogenic enzymes in response to glucose and fructose intake [13, 14]. Our recent studies have shown that ChREBPα protein is significantly downregulated in the livers of mouse models with diet‐induced MASH [15] and CCL4‐induced liver injury/fibrosis [16]. However, how ChREBP⍺ is affected by hepatic stress remains unexamined. Post‐translational modifications are essential for regulating ChREBP⍺ transcriptional activity, subcellular localization, and protein stability in various cell types [17]. In hepatocytes, ChREBP⍺ can be modified through phosphorylation [18], *O*‐glycosylation [19], acetylation [20], and ubiquitination [21, 22]. We previously found that fructose stabilizes ChREBP⍺ by preventing its ubiquitination [21]. However, whether these modifications contribute to the decrease in ChREBP⍺ protein in the context of metabolic and inflammatory stresses remains unexplored.

Ring Finger Protein 8 (RNF8) is a vital E3 ubiquitin ligase involved in the DNA‐damage response to promote DNA repair [23]. RNF8 expression was found to be increased in HCC (hepatocellular carcinoma) tissues and was linked to a poor prognosis in HCC [24]. Acute depletion of *RNF8* in HCC cells impairs epithelial‐mesenchymal transition [25]. RNF8's ubiquitination substrates include H2AX, RecQL4, and NONO, which mainly participate in the DNA damage response [23]. It is unknown whether RNF8 targets metabolic regulators in the liver during MASLD/MASH.

The hepatocyte JNK1/2 pathway is one of the key mediators of insulin resistance and fatty acid‐induced hepatotoxicity [26, 27]. Both oxidative stress and ER stress can strongly activate JNK, leading to JNK‐dependent hepatocyte death [26, 28]. HFD‐induced obesity triggers JNK1/2 phosphorylation and activation in multiple metabolic tissues, especially the liver, in both animal and human studies of obesity [29, 30]. Mouse genetic studies showed that global *JNK1* ^*−/−*^ mice are protected from HFD‐induced liver steatosis and injury compared to WT littermates [31]. Additionally, treatment with the JNK inhibitor SP6000125 improved fatty liver in HFD‐fed rats [32]. These findings suggest that activating the JNK signaling pathway may promote MASLD progression through mechanisms that are not fully understood. JNK controls its cellular activity by directly phosphorylating downstream targets [33, 34], affecting protein degradation, localization, signaling, and interactions with other proteins in a tissue‐ and signal‐specific manner [34]. However, the main substrates of JNK during MASLD progression remain unclear.

Our current study has identified hepatocyte ChREBPα as a cellular stress sensor responding to nutritional and inflammatory stress during MASLD/MASH development. We find that the E3 ligase RNF8 controls ubiquitination and degradation of ChREBPα in hepatocytes upon lipotoxicity/inflammation (PA/TNFα) insult. Inhibition of JNK2 prevents PA/TNFα‐induced and RNF8‐mediated ubiquitination and degradation of ChREBPα in vitro. Lastly, we provide in vivo evidence that knocking down *Rnf8* or *Jnk2* in the liver stabilizes hepatic ChREBPα and reduces MASH induced by the HFLMCD (high‐fructose, low‐methionine, choline‐deficient) diet. Our findings reveal the molecular mechanism of stress‐induced ChREBPα degradation and suggest that targeting RNF8‐mediated ubiquitination of ChREBPα could be a new approach for treating MASH.

## 2 Materials and Methods

### 2.1 Animal Experiments

All animal procedures were approved by the Institutional Animal Care and Research Advisory Committee at the University of Michigan. *C57BL/6* mice were purchased from the Jackson Laboratory and housed under standard 12‐h light/dark cycles with free access to food and water. To induce MASLD, mice were fed HFLMCD diet (Research Diets; A06071309) for designated periods and fasted for 6 h before sacrifice.

### 2.2 Adenovirus Construction

Adenoviruses, including Ad‐GFP, Ad‐Chrebpα‐WT, Ad‐shLacZ, and Ad‐shChrebpα, were generated as previously reported [15]. The targeting sequences for knocking down mouse *Jnk2* and *Rnf8* coding regions are 5′‐GATGCAGCAGTAAGTAGCAA‐3′ and 5′‐GCACCATTACAGGGTTATTGC‐3′, respectively.

### 2.3 Primary Mouse Hepatocyte Isolation and Treatment

Primary mouse hepatocytes (PMHs) were isolated from mice using a previously established protocol [35]. Cells were cultured in serum‐free media overnight before various treatments. Palmitate (300 μM, 24 h) and TNFα (10 ng/mL, 6 h) were used to mimic the MASH condition in vitro. MG132 (5 μM for 16 h to inhibit proteasome), Bafilomycin (100 nM for 16 h to inhibit autophagy), and Cycloheximide (100 ng/mL to inhibit protein synthesis) were used.

### 2.4 Immunoblotting Analysis

Whole‐cell lysates were extracted from primary hepatocytes or frozen liver tissues using RIPA buffer. Protein concentrations were determined using a BCA assay. After SDS‐PAGE, the protein was transferred to nitrocellulose membranes and incubated with a specific antibody. Protein bands were visualized by enhanced chemiluminescence. Anti‐ChREBP (CST#58069); Anti‐Ubiquitin (Sigma#U5379); Anti‐JNK (Santa Cruz #7345); Anti‐RNF8 (Santa Cruz #271462).

### 2.5 Coimmunoprecipitation Assay

Cells were collected in FLAG‐IP buffer supplemented with protease inhibitors and sodium fluoride. Lysates were incubated on ice for 30 min, followed by centrifugation at 15000 × g for 10 min at 4°C to remove debris. The supernatants were collected and incubated overnight at 4°C with gentle rotation using anti‐FLAG M2 Affinity Gel (Millipore‐Sigma A2220). Immunocomplexes were washed 3–5 times with IP buffer, and bound proteins were eluted with SDS loading buffer by heating at 95°C for 10 min. SDS‐PAGE and immunoblotting were used to analyze the eluted proteins.

### 2.6 BODIPY Staining

Primary mouse hepatocytes were plated in 12‐well plates at a density of 8 × 10^4^ cells per well with adenovirus added. After 24 h, the medium was replaced with serum‐free DMEM, followed by treatment with 200 μM palmitate and 400 μM oleic acid for 6 h. The cells were then washed and cultured overnight in fresh serum‐free DMEM. On the third day, the cells were rinsed with PBS and incubated with 2 μM BODIPY 493/503 (Invitrogen) for 15 min. After washing, the cells were mounted and imaged under a fluorescence microscope.

### 2.7 Biochemical Evaluation

Serum alanine transaminase (ALT) and total triglyceride levels were measured using assay kits (Cat# A7525 and A7510, respectively; Pointe Scientific) according to the manufacturer's instructions. For hepatic lipid analysis, liver tissues were weighed and homogenized in 1% acetic acid (7 μL/mg tissue). After brief centrifugation, 200 μL of the supernatant was transferred to a 1.5 mL microcentrifuge tube containing 800 μL of chloroform/methanol (2:1, v/v). After centrifugation at 10 000 × *g* for 10 min at room temperature, 450 μL of the lower organic phase was collected and allowed to evaporate overnight in a chemical fume hood. The dried lipid extracts were dissolved in 200 μL of isopropanol and incubated at 55°C for 20 min. Liver triglyceride levels were measured using 3 μL of the dissolved lipid solution with commercial kits (Cat# 23–666‐410, Pointe Scientific).

### 2.8 RNA Extraction and RT‐qPCR

Total RNA was isolated using TRIzol reagent (Invitrogen) followed by chloroform extraction. First‐strand cDNA synthesis was carried out using the Verso cDNA Synthesis Kit (Thermo Fisher Scientific). Quantitative PCR was performed with Radiant Green 2X qPCR Mix (Alkali Scientific) on an ABI 7900HT Real‐Time PCR System (Applied Biosystems). Relative gene expression levels were calculated using the 2^−ΔΔCt^ method and normalized to the housekeeping gene *18S rRNA* or *Gapdh*. Data were presented as fold change relative to the control. Primer sequences used for qPCR are available upon request.

### 2.9 Picro‐Sirius Red Staining

Paraffin‐embedded liver sections were deparaffinized in xylene and rehydrated through a graded ethanol series (100%, 95%, and 75%). Slides were then stained with 0.1% Picro‐Sirius Red solution for 1 h, followed by two washes in 0.5% acetic acid. Dehydration was carried out using three changes of 100% ethanol. After clearing in xylene, sections were mounted with a resin‐based medium.

### 2.10 Statistical Analysis

Statistical analysis was performed using Prism version 10.0. All results are given as the mean ± SD. Statistical significance was determined either by two‐tailed Student's *t*‐test for comparison between two groups or by one‐way or two‐way ANOVA with Tukey's or Dunnett's post hoc test for multiple group comparison. Results were considered statistically significant with *a* *p*‐value < 0.05.

## 3 Results

### 3.1 Hepatocyte ChREBPα Is Targeted for Ubiquitination and Proteasomal Degradation in Response to Metabolic and Inflammatory Stresses

**FIGURE 1:**
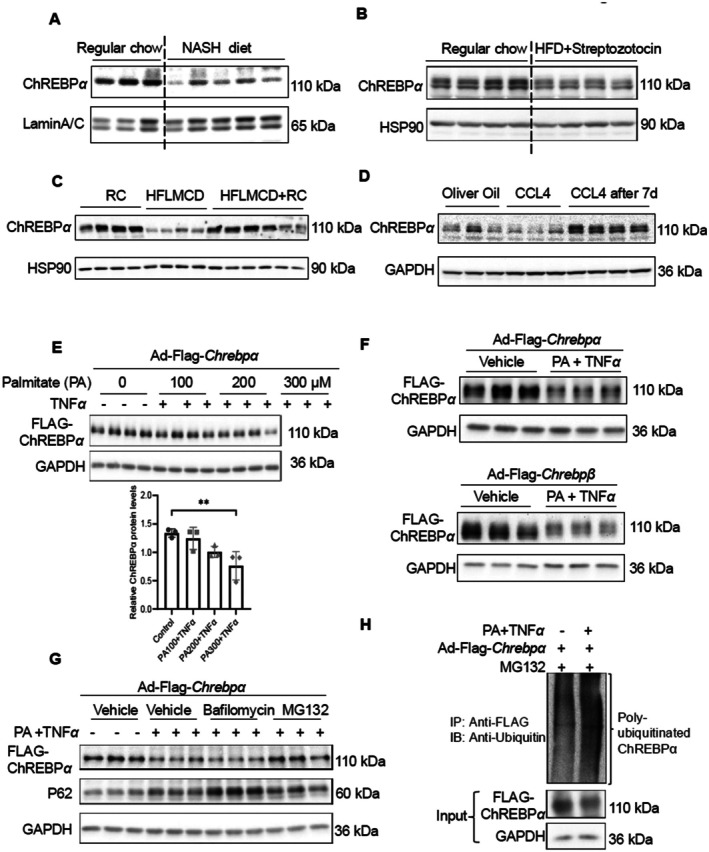
Metabolic/inflammatory stress‐induced ubiquitination and degradation of ChREBPα protein in hepatocytes. (A,B) Liver ChREBPα protein in *C57BL/6* male mice after 20 weeks of NASH diet feeding (*n* = 3, 5) and after 12 weeks of HFD plus streptozotocin injection (40 mg/kg, five consecutive daily injections at the 5th week) (*n* = 4, 4). (C) Liver ChREBPα protein in *C57BL/6* male mice after 9 weeks of HFLMCD diet feeding or 6 weeks of HFLMCD diet feeding followed by 9 weeks of regular chow (*n* = 4, 4, 6). (D) Liver ChREBPα protein in *C57BL/6* male mice immediately or 7 days after 4 weeks of CCL4 injection (*n* = 3, 3, 4). (E) Hepatocyte ChREBPα protein levels and quantification in PMHs treated with TNFα and increasing amounts of palmitate. (F) Hepatocyte ChREBPα and ChREBPb protein levels in PMHs treated with palmitate and TNFα. (G) Hepatocyte ChREBPα protein levels in PMHs treated with palmitate and TNFα, followed by MG132 (5 μM) or Bafilomycin A1 (100 μM) treatment for 16 h. (H) Immunoprecipitation analysis of the ubiquitination of ChREBPα in PMHs treated with palmitate and TNFα, followed by MG132 treatment for 6 h. Data are representative of at least three independent experiments. Data are plotted as mean ± SD. **p‐value < 0.01 by Student's *t*‐test for C and by one‐way ANOVA with post hoc test for panel E.

To assess the impact of hepatic stress on ChREBPα protein expression, we first examined hepatic ChREBPα protein expression in mice fed a 20‐week NASH diet and in mice on a 12‐week HFD combined with streptozotocin injection, which creates an advanced diabetic model [36]. In both cases, hepatic ChREBPα protein levels were significantly decreased (Figure 1A,B). To further investigate the relationship between hepatic ChREBPα levels and the degree of hepatic stress, we generated two liver fibrosis resolution models, including HFLMCD diet‐induced MASH and recovery with chow diet, and CCL4‐induced liver fibrosis and recovery from hepatotoxin. In both cases, hepatic ChREBPα levels were markedly downregulated by either diet or CCL4 but recovered to almost normal levels when the stress was removed (Figure 1C,D). These findings suggest that hepatic ChREBPα levels are negatively associated with hepatic stress and that downregulation of ChREBPα could be an indicator of the hepatic stress response.

Lipotoxicity and excessive inflammation are common stress signals during MASH. We hypothesize that the downregulation of ChREBPα protein may be a direct result of hepatocyte stress responses. To simulate this stress response in vitro, we treated primary mouse hepatocytes (PMHs) with a cocktail of palmitate (PA) and TNFα to replicate the intrahepatic environment of MASH [37, 38]. Although the mRNA levels of ChREBPα were unaffected by the co‐treatment of PA and TNFα (Figure S1A), the ChREBPα protein was degraded in a dose‐dependent manner upon PA plus TNFα treatment (Figure 1E). Treatment of PMHs with 300 μM PA and 10 ng/mL TNFα reduced ChREBPα protein levels by over 50%. Notably, PA alone was insufficient to decrease ChREBPα protein. Conversely, TNF‐α alone reduced ChREBPα protein but not as effectively as the combined PA and TNF‐α treatment (Figure S2). Therefore, we used the PA/TNF‐α cocktail to induce ChREBPα protein degradation throughout the rest of the study. We also observed that this response was specific to PA plus TNFα treatment, as other MASH‐relevant cytokines, including IL‐1β, IL‐18, and IL‐33, did not impact ChREBPα protein levels (Figure S3). MASH is also known to be associated with oxidative stress [9], unresolved endoplasmic reticulum stress [10], and mitochondrial stress [29]. To test if hepatocyte ChREBPα is also impacted by these stress signals, we treated primary hepatocytes with H_2_O_2_ to induce oxidative stress, Tunicamycin to induce ER stress [39], and FCCP to induce mitochondrial stress [40]. Our results showed that hepatocyte ChREBPα protein abundance is reduced by H_2_O_2_ but not Tunicamycin or FCCP (Figure S3B). Taken together, we have identified at least two MASH‐associated stress signals that could potently reduce ChREBPα protein abundance in hepatocytes.

ChREBPα protein consists of the N‐terminal regulatory domain, also called LID (Low‐Glucose Inhibitory Domain), NLS/NES (Nuclear Localization and Export Signals), and the C‐terminal DNA‐binding region. The ChREBPβ isoform, an alternative splicing variant of ChREBPα [41], lacks the 177 amino acids of the N‐terminus, the major portion of LID. We found that ChREBPβ exhibited a similar response to PA/TNFα (Figure 1F), indicating that both ChREBP isoforms share a common regulatory mechanism at the level of post‐translational modifications, which is independent of the N‐terminal LID motif.

ChREBPα protein is known to be regulated by ubiquitination and proteasome degradation [17]. We first tested whether PA/TNFα‐induced ChREBPα degradation was proteasome‐dependent. We treated cells with either Bafilomycin (an autophagy inhibitor) or MG132 (a proteasome inhibitor) after PA/TNFα treatment. Both Bafilomycin and MG132 increased the protein levels of p62, a known target of autophagy. Only MG132 restored ChREBPα protein levels in cells treated with PA/TNF (Figure 1G). Moreover, PA plus TNFα increased the formation of ChREBPα‐ubiquitin conjugates in PMHs (Figure 1H), suggesting that combined metabolic and inflammatory stresses stimulate ChREBPα ubiquitination and promote its degradation.

### 3.2 RNF8 Acts as a Novel E3 Ligase for Degrading ChREBPα in Hepatocytes

**FIGURE 2:**
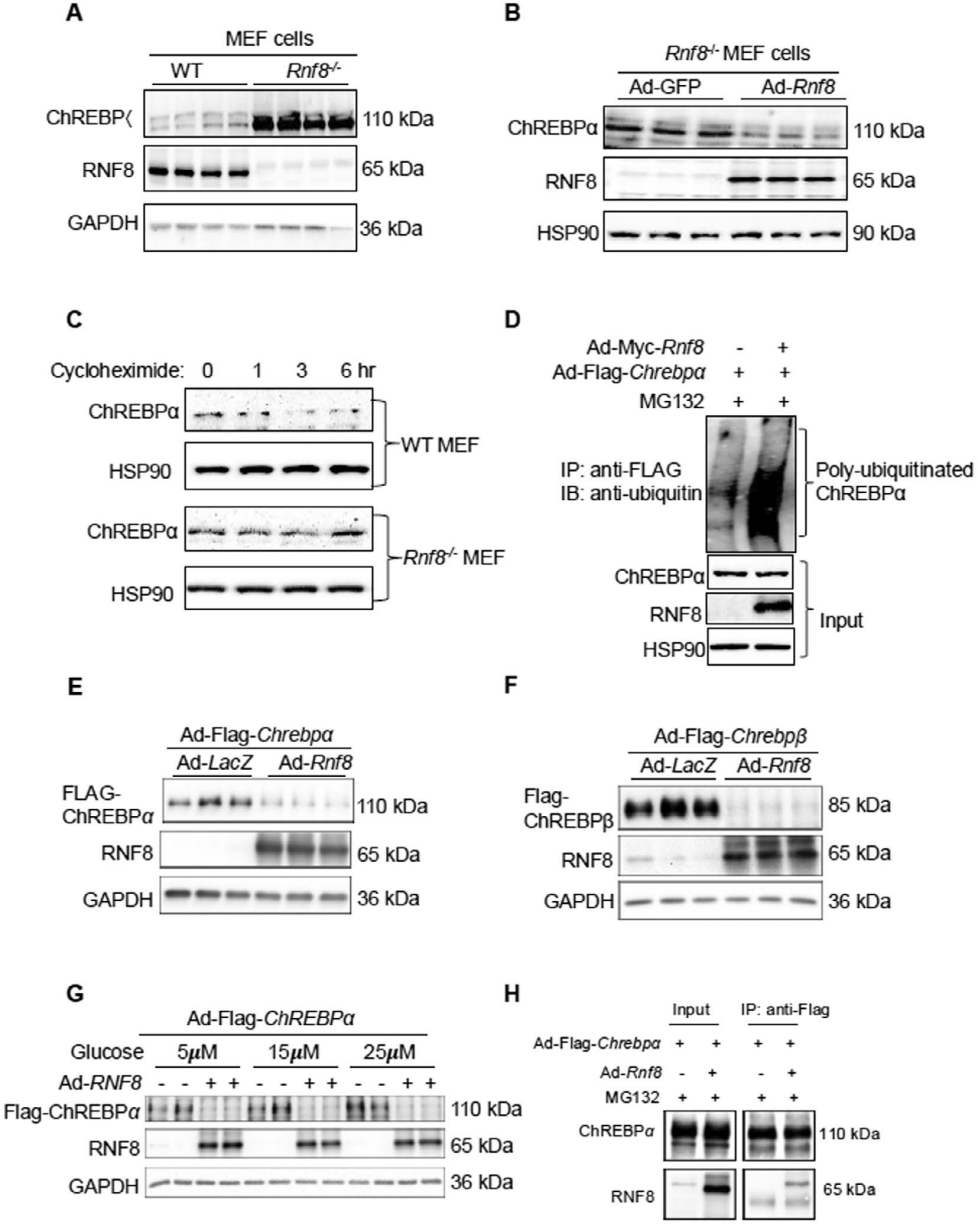
RNF8 mediates the ubiquitination and degradation of ChREBPα in hepatocytes. (A) Protein abundance of exogenous ChREBPα in WT vs. *Rnf8* ^−/−^ MEF cells. (B) Protein abundance of exogenous ChREBPα in Rnf8−/− MEF cells transduced with Ad‐GFP or Ad‐Rnf8. (C) Protein half‐life of ChREBPα in WT vs. *Rnf8* ^−/−^ MEF cells treated with cycloheximide for 0–6 h; (D) Enhanced ChREBPα ubiquitination upon RNF8 overexpression in *Rnf8* ^*−/−*^ MEF cells. (C) Protein levels of ChREBPα and RNF8 in PMHs treated with palmitate (300 μM, 24 h) and TNFα (10 ng/mL, 6 h). (D) Protein levels of ChREBPα and RNF8 in PMHs transduced with Ad‐Flag‐*Chrebpα* and either Ad‐*LacZ* or Ad‐*Rnf8*. (E,F) Protein levels of exogenous ChREBPα and ChREBPβ in PMHs transduced with either Ad‐lacZ or Ad‐Rnf8. (G) Protein levels of exogenous ChREBPα in PMHs transduced with Ad‐Rnf8 and then treated with increasing doses of glucose (5–25 μM) for 16 h. (H) Immunoprecipitation/immunoblotting analysis showed that ChREBPα interacts with RNF8 in PMHs treated with MG312.

We hypothesized that PA/TNFa induces ubiquitinataion and degradation of ChREBPα depending on a specific E3 ligase. Thus, we focused on E3 ligases which have been shown to play a role in general stress response [42, 43]. Using targeted screening methods (data not shown), we identified the Ring‐Finger 8 (RNF8) E3 ligase as a novel E3 ligase that may mediate ChREBPα degradation for a number of reasons. At first, RNF8 is known to play a critical role in DNA damage repair upon genotoxic stress. Secondly, ChREBPα protein levels were significantly higher in *Rnf8* ^*−/−*^ MEF cells (Figure 2A) [44], whereas restoring RNF8 expression in *Rnf8* ^*−/−*^ MEFs reduced ChREBPα protein levels and increased ChREBPα‐ubiquitin conjugates (Figure 2B,C). Moreover, the degradation rate of ChREBPα was considerably slower in *Rnf8* ^*−/−*^ MEFs compared to WT‐MEFs (Figure 2D). Lastly, RNF8 overexpression is sufficient to degrade both ChREBPα and ChREBPβ in mouse primary hepatocytes (Figure 2E,F), and enhance the degradation rate of ChREBPα (Figure S4).

To examine whether the C‐terminal DNA‐binding domain of ChREBPα is necessary for its response to PA/TNFa, we generated two C‐terminal deletion mutants, Chrebpα 1‐200aa and 1‐400aa, and found that although ChREBPα full‐length and 1‐400aa are reduced by RNF8 overexpression, ChREBPα 1‐200aa is unaffected by RNF8 (Figure S5). Together, our data suggest that RNF8 targets the region of ChREBPα between 200aa and 400aa.

We also examined the impact of high glucose on RNF8‐driven ChREBPα degradation, as high glucose has been reported to enhance ChREBPα activity and stability through *O‐*glycosylation [19]. Our data show that RNF8 promotes ChREBPα degradation regardless of glucose levels (Figure 2G). Interestingly, we were able to capture the protein–protein interaction between ChREBPα and RNF8 in hepatocytes treated with MG132 (Figure 2H), supporting that RNF8 needs to bind to ChREBPα prior to ubiquitination. Taken together, these results support that RNF8 promotes ChREBPα ubiquitination and degradation in both MEF and mouse hepatocytes.

### 3.3 RNF8 Is Required for Metabolic/Inflammatory Stress‐Induced Degradation of ChREBPα in Mouse Hepatocytes

**FIGURE 3:**
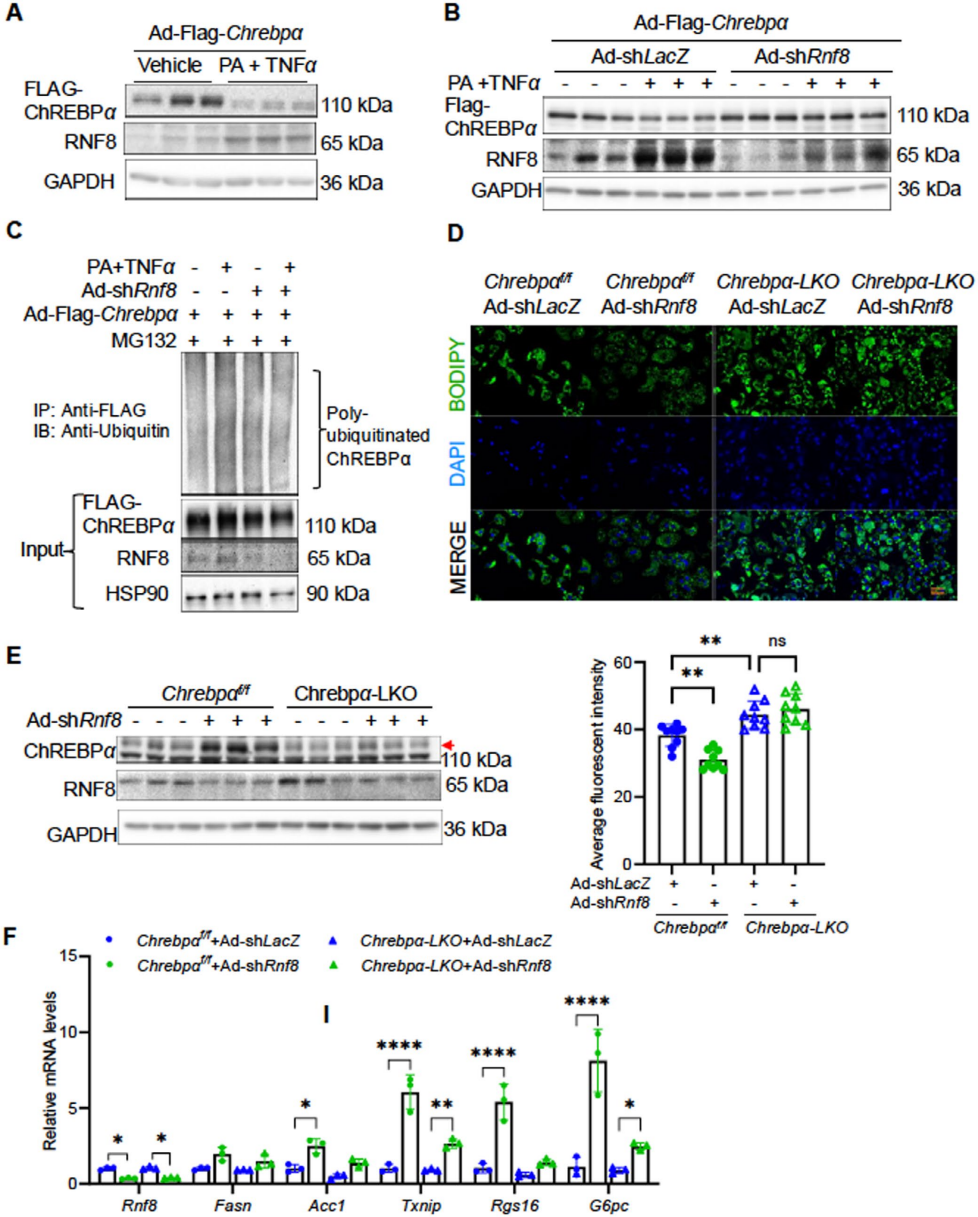
RNF8 is required for metabolic/inflammatory stresses‐induced ChREBPα degradation. (A) Protein levels of ChREBPα and RNF8 in PMH treated with palmitate (300 μM, 24 h) and TNFα (10 ng/mL, 6 h). (B) Protein levels of ChREBPα and RNF8 in PMHs transduced with Ad‐Flag‐*Chrebpα* and either Ad‐sh*LacZ* or Ad‐sh*Rnf8*, followed by treatment with palmitate and TNFα. (C) Immunoprecipitation analysis of ChREBPα ubiquitination in PMH transduced with Ad‐Flag‐*Chrebpα* and either Ad‐sh*LacZ* or Ad‐sh*Rnf8*, treated with palmitate and TNFα, then with MG132 for 6 h. (D) PMHs were isolated from *Chrebp* ^*flox/flox*^ and *Chrebpα‐LKO mice and* transduced with either Ad‐sh*LacZ* or Ad‐sh*Rnf8* before BODIPY staining. (E,F) Immunoblot analysis of ChREBPα, RNF8, and qRT‐PCR of ChREBPα target genes in PMHs from *Chrebpα* ^*flox/flox*^ and *Chrebpα‐LKO mice*, transduced with either Ad‐sh*LacZ* or Ad‐sh*Rnf8*. Data are representative of at least three independent experiments. Results are presented as mean ± SD. *p‐value < 0.05, **p‐value < 0.01, and ****p‐value < 0.0001, using two‐way ANOVA with post hoc Tukey's test for panel D and F.

To further investigate whether RNF8 serves as a stress‐related E3 ligase for ChREBPα in hepatocytes, we first assessed RNF8 expression in mouse primary hepatocytes challenged with PA/TNFα. PA/TNFα reduced ChREBPα levels but elevated endogenous RNF8 (Figure 3A). Next, we generated *adshRnf8* and used it to acutely deplete endogenous Rnf8 expression prior to PA/TNFα stimulation. Under these conditions, depletion of RNF8 abrogated PA/TNFα‐induced ubiquitination and degradation of ChREBPα in cultured hepatocytes (Figure 3B,C). Taken together, these findings support the idea that RNF8, in addition to sensing genotoxic stress, responds to metabolic and inflammatory stresses. Under such conditions, ChREBPα is a direct target of RNF8 in hepatocytes.

To date, we have shown that *Rnf8* depletion stabilizes the ChREBPα protein. Whether RNF8 deletion affects ChREBPα function remains to be tested. Thus, we examined whether RNF8 plays a role in ChREBPα‐dependent gene expression. Both WT and *Chrebpα‐LKO* PMHs were transduced with Ad‐shLacZ or Ad‐shRnf8. Acute depletion of hepatic *Rnf8* increased ChREBPα protein levels in WT hepatocytes but not in *Chrebpα‐LKO* cells (Figure 3D). At the mRNA level, *Rnf8* depletion elevated expression of several ChREBPα targets, including *Acc1, Txnip, Rgs16*, and *G6pc*, in WT but not in *Chrebpα‐LKO* hepatocytes (Figure 3E). We previously reported that ChREBPα overexpression protects hepatocytes from lipid overload [15]. Here, we observed that acute depletion of *Rnf8* reduced the number of lipid droplets detected by BODIPY staining in a ChREBPα‐dependent manner in PMHs (Figure 3F,G). In summary, RNF8 emerges as a new lipid regulator within hepatocytes by degrading ChREBPα.

### 3.4 Depletion of Hepatic RNF8 Restores ChREBPα Levels and Protects Mice From HFLMCD‐Induced Liver Injury, Steatosis, and Inflammation

**FIGURE 4:**
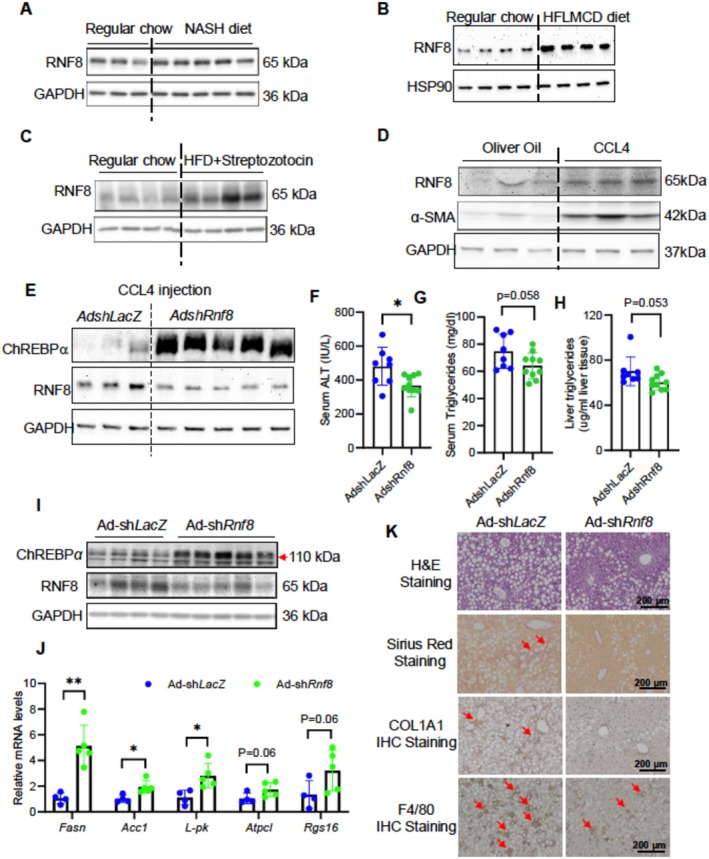
Acute depletion of hepatic RNF8 restores ChREBPα protein and partially attenuates HFLMCD diet‐induced liver injury, steatosis, and inflammation in mice. (A‐B) Liver RNF8 protein in *C57BL/6* male mice after 20 weeks of NASH diet feeding (*n* = 3, 5) and after 9 weeks of HFLMCD diet feeding (*n* = 4,4). (C) Liver ChREBPα protein in *C57BL/6* fed with 12 weeks HFD plus streptozotocin injection (40 mg/kg, five consecutive daily injections at the 5th week) (*n* = 4, 4). (D) Liver ChREBPα protein in *C57BL/6* male mice immediately or 7 days after 4 weeks of CCL4 injection (*n* = 3, 3). (E–J) 10‐week‐old wildtype male mice were injected with *Ad‐shLacZ v.s Ad‐shRnf8* and fed an HFLMCD diet for 10 days before dissection. (E) Liver protein levels of ChREBPα and RNF8 in Ad‐sh*LacZ* (*n* = 3) or Ad‐sh*Rnf8 (n = 5)*‐injected mice following CCL4 treatment. (F–H) Serum ALT, serum, and liver triglycerides in Ad‐sh*LacZ* or Ad‐sh*Rnf8*‐injected mice. (H) Liver protein levels of ChREBPα and RNF8 in Ad‐sh*LacZ* versus Ad‐sh*Rnf8* groups. (I) Liver mRNA levels of ChREBPα targets in Ad‐sh*LacZ* versus Ad‐sh*Rnf8* groups. (J) Liver histology showing H&E staining, Sirius Red staining, COL1A1, and F4/80 IHC staining results in Ad‐sh*LacZ* versus Ad‐sh*Rnf8*. Data are representative of at least three independent experiments. Results are plotted as mean ± SD. *p‐value < 0.05 and **p‐value < 0.01 by Student's *t*‐test.

So far, we have gathered strong in vitro evidence that RNF8 is a key negative regulator of ChREBPα protein in response to metabolic and inflammatory stresses in mouse hepatocytes. However, the significance of this regulation in mouse liver, particularly regarding ChREBPα protein stability and liver metabolic health, has not yet been tested. We first examined whether RNF8 levels are altered in chronically stressed liver tissues. Indeed, RNF8 protein levels increased in the livers of mice fed a 20‐week NASH diet (Figure 4A), a 5‐week HFLMCD diet (Figure 4B), or subjected to HFD/STZ treatment (Figure 4C). Moreover, RNF8 protein levels were markedly increased by CCL4, a well‐established hepatotoxin (Figure 4D). These in vivo findings support the in vitro result that RNF8 is an inducible sensor of chronic hepatic stress, regardless of etiology.

Whether RNF8 is involved in regulating endogenous ChREBPα protein levels remains unknown. To investigate this, we first used *Ad‐shRnf8* to acutely deplete hepatic RNF8 protein in mice treated with CCL4. At the end of the experiment, we observed that ChREBPa protein levels were significantly increased in mice injected with *Ad‐shRnf8* (Figure 4E), supporting the idea that RNF8 suppresses ChREBPα protein expression in the mouse liver in the context of CCL4 injection.

Next, we fed mice a high‐fructose, low‐methionine, choline‐deficient (HFLMCD) diet, which is known to induce early‐onset MASH characterized by liver steatosis, mild inflammation, and fibrosis. Mice [15, 45]. Mice received injections of Ad‐sh*LacZ* or Ad‐sh*Rnf8* and were then fed the HFLMCD diet for 10 days before sacrifice. At the end of the experiment, acute depletion of hepatic *Rnf8* significantly lowered serum ALT levels and reduced both serum and hepatic triglycerides, although these differences did not reach statistical significance (Figure 4F–H). Ad‐shRnf8 increased overall ChREBPα protein levels (Figure 4I) and mRNA levels of several classic ChREBPα targets, including *Fasn, Acc1, and L‐pk*, in the liver (Figure 4J). Additionally, liver H&E staining revealed fewer lipid droplets, and F4/80 staining showed decreased macrophage infiltration and hepatic inflammation in mice injected with Ad‐shRnf8. Simultaneously, markers for liver fibrosis, such as Sirius Red staining for collagen and IHC for COL1A1, were also reduced in mice treated with Ad‐shRnf8 (Figure 4K). Collectively, these findings demonstrate that blocking RNF8‐mediated degradation of ChREBPα in the liver can deter the development of HFLMCD diet‐induced MASH.

### 3.5 JNK2 Is Required for RNF8‐Mediated Degradation of ChREBPα in Hepatocytes

**FIGURE 5:**
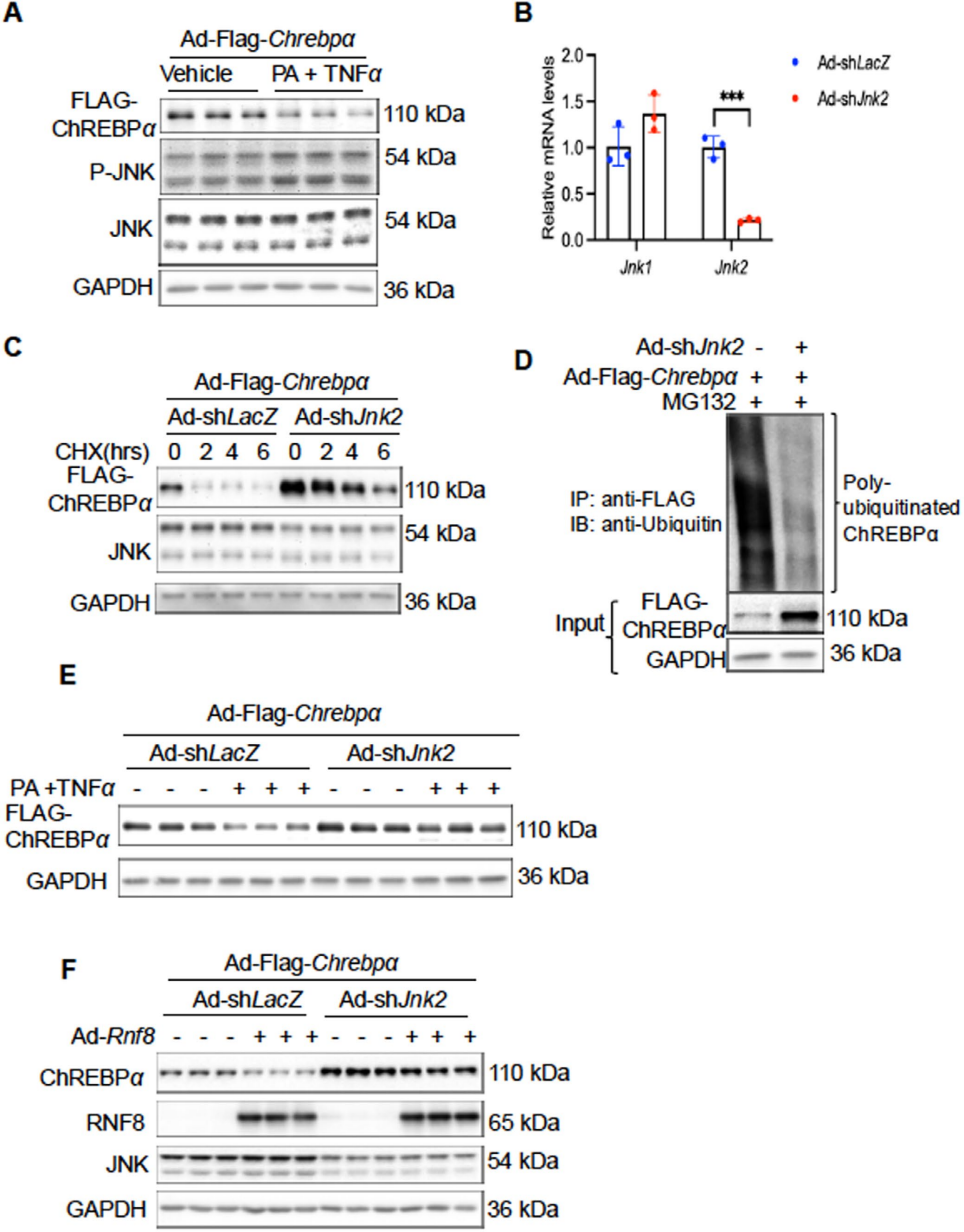
JNK2 is required for RNF8‐mediated degradation of ChREBPα upon metabolic/inflammatory stresses. (A) Immunoblot analysis of ChREBPα, JNK, and phospho‐JNK proteins in PMHs treated with palmitate (300 μM, 24 h) and TNFα (10 ng/mL, 6 h). (B) The mRNA levels of *Jnk1* and *Jnk2* in PMHs transduced with Ad‐shLacZ versus Ad‐shJnk2; (C) Hepatocyte ChREBPα protein levels and quantification in PMHs transduced with Ad‐sh*Jnk2*, followed by cycloheximide (CHX) (100 μg/mL) treatment for 0, 2, 4, and 0, 2, 4, 6 h; (D) Immunoprecipitation analysis of the ubiquitination of ChREBPα in PMHs treated with Ad‐sh*Jnk2*, followed by MG132 treatment for 6 h; (E) Hepatocyte ChREBPα protein levels in PMHs transduced with Ad‐sh*Jnk2* followed by treatment with palmitate and TNFα. (F) Hepatocyte ChREBPα protein levels in PMHs transduced with Ad‐sh*Jnk2* in the presence or absence of AdRnf8. The data are plotted as mean ± SD. *p‐value < 0.05, **p‐value < 0.01, and ****p‐value < 0.0001 by the Student's *t*‐test.

In the context of the DNA damage response, RNF8 coordinates with ATM (Ataxia‐Telangiectasis Mutated) to degrade its target [46]. We hypothesize that in response to metabolic or inflammatory stresses, RNF8 also works with a specific kinase to promote ubiquitination and degradation of ChREBPα. Sustained JNK activation has been linked to the development of liver diseases such as MASLD/MASH, drug‐induced liver injury, and alcoholic liver disease [5, 11]. Various stress signaling molecules are known to activate JNK within hepatocytes, including high doses of free fatty acids [26], H_2_O_2_ [47], and inflammatory cytokines [48]. So far, it has not been examined whether JNK activation contributes to RNF8‐mediated ChREBPα degradation following PA/TNFα stimulation. In fact, PA/TNFα induces JNK phosphorylation while decreasing the levels of ChREBPα protein in cultured hepatocytes (Figure 5A). To determine whether JNK activation is involved in ChREBPα degradation caused by PA and TNFα, we created shRNA targeting *Jnk2*, since JNK2, compared with JNK1, is more studied in the context of hepatocyte lipotoxicity and inflammation [31]. We verified the specificity and efficiency of shJnk2 knockdown by RT‐qPCR (Figure 5B). Since the JNK antibody detects both JNK1 and JNK2, it has been difficult to see a clear reduction of JNK2 levels via immunoblotting in cells transduced with Ad‐shJnk2. We verified knockdown efficiency with RT‐qPCR for the rest of the studies.

To gain insights into the role of JNK2 in ChREBPα proteolysis, we examined the protein half‐life of ChREBPα with and without JNK2 depletion in a cycloheximide chase study. As shown in Figure 5C, ChREBPα degraded with a half‐life of about 2 h. However, when *JNK2* was depleted, ChREBPα's half‐life was extended to 4 h. Additionally, *JNK2* depletion caused a significant reduction in ChREBPα‐ubiquitin conjugates, indicating suppressed ChREBPα ubiquitination in PMHs (Figure 5D). More importantly, we found that depletion of JNK2 is sufficient to block RNF8‐induced ChREBPα degradation in hepatocytes (Figure 5E) as well as.

PA/TNFα‐induced ChREBPα degradation (Figure 5F), suggesting that JNK2 in hepatocytes is indeed necessary for degrading ChREBPα protein under RNF8 overexpression or PA/TNFα stress.

### 3.6 Acute Depletion of Hepatic Jnk2 Stabilizes ChREBPα Protein and Improved Liver Metabolism and Health in Mice With HFLMCD Diet

**FIGURE 6:**
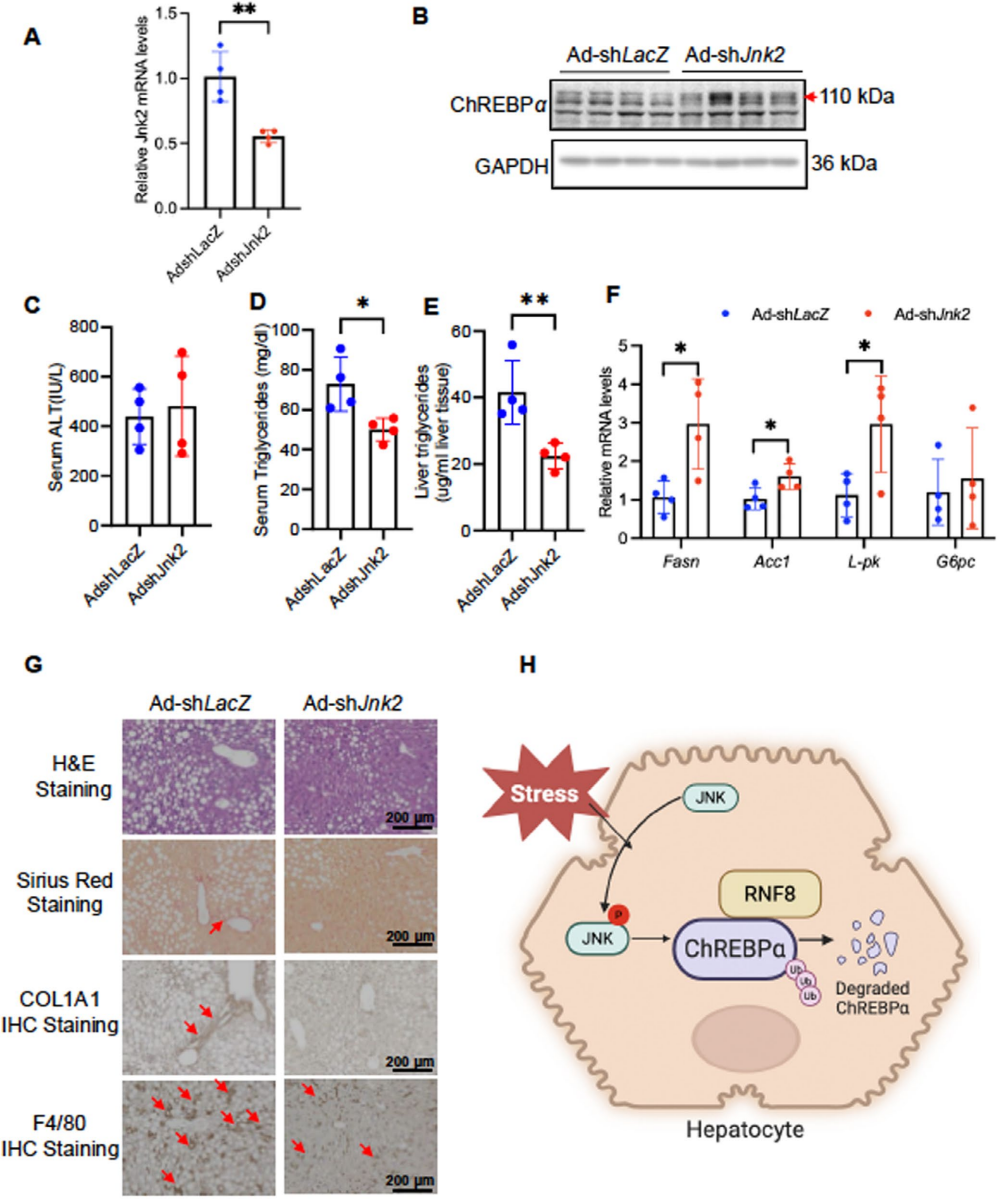
Acute depletion of hepatic Jnk2 restores ChREBPα protein and partially attenuates HFLMCD diet‐induced liver steatosis and inflammation in mice. 10‐week‐old wildtype male mice were injected with Ad‐sh*LacZ v.s* Ad‐shJnk2 and fed an HFLMCD diet for 10 days before dissection. (A) The mRNA levels of hepatic Jnk2 in mice injected with AdshLacZ vs. AdshJnk2 (*n* = 4,4). (B) Liver protein levels of ChREBPα in Ad‐sh*LacZ* versus Ad‐sh*Jnk2* groups. (C‐D) Serum ALT, serum, and liver triglyceride levels in Ad‐sh*LacZ* versus Ad‐sh*Jnk2*. (F) Liver mRNA levels of ChREBPα targets in Ad‐sh*LacZ* versus Ad‐sh*Jnk2* groups. (G) Liver histology with H&E staining, Sirius Red staining, COL1A1, and F4/80 IHC staining in Ad‐sh*LacZ* versus Ad‐sh*Jnk2* groups. (H) The proposed working model: Hepatocytes respond to chronic metabolic and inflammatory stresses by activating JNK2‐RNF8‐mediated proteolysis. One of the downstream targets of the JNK2‐RNF8 stress pathway is ChREBPα. Degradation of ChREBPα is correlated with the progression of MASH. Data are representative of at least three independent experiments. Results are plotted as mean ± SD. *p‐value < 0.05 and **p‐value < 0.01 by Student's *t*‐test.

To further test whether JNK2 is also required for degrading hepatic ChREBPα in stressed mouse liver, we fed mice with HFLMCD for 5 weeks to induce early‐onset MASH and then injected AdshJnk2 to reduce hepatic JNK pathway activity. By the end of the experiment, Jnk2 mRNA in mouse liver was reduced by more than 50% in the AdshJnk2 group compared with Adsh*LacZ* (Figure 6A). In contrast, liver ChREBPα protein levels and ChREBPα targets (*Fasn, Acc1, and L‐pk*) increased upon AdshJnk2 (Figure 6B,C), consistent with our in vitro observations. In the meantime, acute depletion of hepatic *Jnk2* significantly reduced both serum and liver triglycerides without affecting serum ALT levels in WT mice (Figure 6D–F). Additionally, liver H&E staining showed a decrease in lipid droplets, while F4/80 staining indicated reduced macrophage infiltration and hepatic inflammation. In the same liver, markers of liver fibrosis (Sirius Red staining and COL1A1 IHC) were also decreased in mice injected with Ad‐shJnk2 (Figure 6G). Overall, these findings demonstrate that preventing stress‐induced ChREBPα degradation by depleting Rnf8 or *Jnk2* helps alleviate HFLMCD diet‐induced MASH (Figure 6H).

## 4 Discussion

As the most common chronic liver disease worldwide, MASLD/MASH is strongly linked to hepatic lipotoxicity, inflammation, and insulin resistance. During progression from MASLD to MASH, persistent activation of hepatic stress pathways plays a significant role in disease development; however, the specific downstream targets within hepatocytes remain unclear. Here, we present evidence that, in response to metabolic/inflammatory stresses (PA/TNFα), the stress‐induced E3 ligase RNF8 promotes degradation of the ChREBPα protein in hepatocytes in a JNK2‐dependent manner. Depleting either *Jnk2* or *Rnf8* not only stabilizes ChREBPα protein at baseline but also prevents its degradation upon PA/TNFα treatment. In vivo, in a mouse model of diet‐induced MASH, adenovirus‐mediated knockdown of *Jnk2 or Rnf8* increases hepatic ChREBPα protein levels while reducing MASH‐associated liver injury, fatty liver, and inflammation. Therefore, our work suggests that targeting RNF8, which mediates ChREBPα ubiquitination and degradation in the liver, may be a new strategy for treating MASLD/MASH.

Our work highlights RNF8, a key factor in DNA double‐strand break signaling [23, 46], as a new player in the liver's nutritional stress response. Its protein levels are increased in the livers of mouse models with either nutritional/metabolic stress or exposure to the hepatotoxin CCL4. So far, very little is known about how nutritional, hormonal, and inflammatory signals regulate hepatic RNF8 protein levels. Interestingly, RNF8 can catalyze its own ubiquitination, which can lead to its degradation if not balanced by deubiquitinating enzymes. For example, the deubiquitinating enzyme ATAXIN3 is found to interact with RNF8 and deubiquitinate RNF8 upon genotoxic stress [49]. It has been found that stress such as proteotoxicity and oxidative stress profoundly induces the activity and subcellular location of the ATAXIN3 protein [50, 51]. Future work is warranted to investigate the potential role of ATAXIN3 in the hepatocyte stress response, especially on the RNF8‐mediated pathway.

We demonstrated that RNF8's metabolic effects on lipid overload‐induced lipid droplet formation and lipogenic gene expression are mediated through hepatocyte ChREBPα, establishing a new nutrient‐sensing pathway triggered by RNF8. Moreover, acute depletion of hepatic RNF8 alleviates diet‐induced liver steatosis, fibrosis, and inflammation. This finding is somewhat surprising, since the canonical action of RNF8 is to regulate the DNA damage response. Our work expands current knowledge of RNF8 as a novel metabolic regulator, especially during the pathogenesis of diet‐induced liver diseases. It is very likely that RNF8 may also target other metabolic proteins in addition to ChREBPα in the liver. We found that RNF8 degrades ChREBPα in stressed hepatocytes in a JNK2‐dependent manner. Whether this mechanism affects other RNF8 substrates remains to be explored.

One of our key findings shows that JNK2‐dependent stress responses regulate hepatocyte ChREBPα. To our knowledge, this is the first evidence linking ChREBPα, a well‐known lipogenic transcription factor, to the hepatic stress response pathway. The biochemical basis by which JNK2 coordinates with RNF8 to degrade ChREBPα upon PA/TNFα stimulation remains unclear. Given that JNK regulates the ubiquitination and degradation of its substrates through site‐specific phosphorylation [33, 52], we hypothesized that JNK2 might target ChREBPα for phosphorylation‐dependent degradation. Using Scansite 4 (scansite4.mit.edu) and PhosphoSitePlus (www.Phosphosite.org), we identified a phosphorylation motif centered on T311 in mouse ChREBPα, which shows a high degree of similarity to other known JNK targets, including ITCH, IRS‐1, and JIP1 [52]. Because of the lack of phosphor‐specific antibodies that detect phosphorylated ChREBPα in vivo, we cannot definitively confirm that JNK2 directly phosphorylates ChREBPα upon PA/TNFα stimulation in vivo.

In summary, as a stress sensor, hepatocyte ChREBPα degradation is triggered by metabolic and inflammatory stresses and requires the coordinated action of JNK2 and RNF8. This suggests that targeting the JNK2‐RNF8 pathway involved in ChREBPα ubiquitination and degradation could be a promising strategy for treating MASH. However, we are fully aware of several limitations in our current study. Our study focused on how the mouse ChREBPα protein responds to hepatocyte stress signals. Whether the human ChREBPα protein is regulated in a similar manner remains to be tested, especially in human MASH patients. Moreover, the long‐term effects of stabilizing ChREBPα on diet‐induced MASH remain unknown. Nonetheless, our study uncovers a novel biochemical mechanism behind stress‐induced degradation of ChREBPα. It indicates that targeting the JNK2‐RNF8 pathway involved in ChREBPα ubiquitination and degradation could be a promising strategy for treating MASH.

## Author Contributions

Y.Z. with help from S.W., J.Z., and G.Z. carried out all the animal experiments, performed tissue analysis, analyzed the data and generated figures. Y.Z. performed all the biochemical analysis with help from Q.Z., Y.Z., S.W., J.O, and Z.Z. generated all the expression vectors and recombinant adenoviruses for in vitro and in vivo experiments with guidance from X.T. L.Y. and X.T. supervised the project and wrote the manuscript with help from Y.Z. Inputs and suggestions from all authors were incorporated.

## Funding

This work was supported by HHS | NIH | NIDDK | Division of Diabetes, Endocrinology, and Metabolic Diseases (DEM) (Grants DK099593 and DK121170).

## Conflicts of Interest

The authors declare no conflicts of interest.

## Supporting information


**Figure S1:** Combined treatment with Palmitate and TNFα does not affect Chrebpα mRNA levels in primary mouse hepatocytes. Cells were treated with Palmitate (300 μM, 24 h) and TNFα (10 ng/mL, 6 h) before being harvested for RNA extraction and mRNA analysis by QPCR. Data are presented as mean ± SD.
**Figure S2:** Immunoblot analysis of ChREBPα expression in PMHs transduced with Ad‐Flag‐*ChREBPα* and then treated with palmitate (300 μM, 24 h) alone (A), TNFα (10 ng/mL, 6 h) alone (B), or palmitate (300 μM, 24 h) plus TNFα (10 ng/mL, 6 h) (C). ChREBPα protein levels were quantified by *Image J* (D). The data were plotted as mean ± SD. * *p*‐value < 0.05, ** *p*‐value < 0.01 by the Student's t‐test.
**Figure S3:** (A) Effects of common pro‐inflammatory cytokines on ChREBPα protein in hepatocytes. PMHs were transduced with Ad‐Chrebpα and treated with palmitate (300 μM) and a cytokine—either IL‐1β (15 ng/mL), IL‐18 (5 ng/mL), or IL‐33 (50 ng/mL). (B‐D) Effects of MASH‐associated stress signals on ChREBPα protein levels in hepatocytes. PMHs were transduced with Ad‐Chrebpα and treated with H_2_O_2_ (400 μM), tunicamycin (2 μM), and FCCP (2 μM) for 6 h before harvest. The protein levels of ChREBPα were examined by immunoblotting with anti‐ChREBPα.
**Figure S4:** RNF8 accelerates the degradation rate of ChREBPα in mouse hepatocytes. Cells were first transduced with Ad‐ChREBPα with or without Ad‐RNF8 before treatment with Cycloheximide (CHX 100 μg/mL) for the indicated times. The relative expression of ChREBPα protein was normalized to the loading control GAPDH. The ChREBPα level at 0 h time point represents 100%. Data are presented as mean ± S.D. from three independent experiments.
**Figure S5:** Effects of C‐terminal deletion of ChREBPα protein on response to RNF8‐mediated degradation; 293 Ad cells were first transfected with expression vectors encoding either Flag‐Chrebpα full‐length, 1‐200aa, or 1‐400aa truncation mutants prior to treatment with Ad‐Rnf8. The cells were then harvested for immunoblotting to examine the abundance of ChREBPα protein using anti‐Flag.

## Data Availability

Included in article.
