# Supplementary material for: The E3 Ligase RNF8 Promotes Ubiquitination and Degradation of ChREBPα During Liver Stress Response
Source: FASEB J. 2026 Apr 29;40:e71830. doi: 10.1096/fj.202600106R (PMC13126098; doi:10.1096/fj.202600106R)
Supplement: Supplementary file 1 — Figure S1: Combined treatment with Palmitate and TNFα does not affect Chrebpα mRNA levels in primary mouse hepatocytes. Cells were treated with Palmitate (300 μM, 24 h) and TNFα (10 ng/mL, 6 h) before being harvested for RNA extraction and mRNA analysis by QPCR. Data are presented as mean ± SD. Figure S2: Immunoblot analysis of ChREBPα expression in PMHs transduced with Ad‐Flag‐ChREBPα and then treated with palmitate (300 μM, 24 h) alone (A), TNFα (10 ng/mL, 6 h) alone (B), or palmitate (300 μM, 24 h) plus TNFα (10 ng/mL, 6 h) (C). ChREBPα protein levels were quantified by Image J (D). The data were plotted as mean ± SD. * p‐value < 0.05, ** p‐value < 0.01 by the Student's t‐test. Figure S3: (A) Effects of common pro‐inflammatory cytokines on ChREBPα protein in hepatocytes. PMHs were transduced with Ad‐Chrebpα and treated with palmitate (300 μM) and a cytokine—either IL‐1β (15 ng/mL), IL‐18 (5 ng/mL), or IL‐33 (50 ng/mL). (B‐D) Effects of MASH‐associated stress signals on ChREBPα protein levels in hepatocytes. PMHs were transduced with Ad‐Chrebpα and treated with H2O2 (400 μM), tunicamycin (2 μM), and FCCP (2 μM) for 6 h before harvest. The protein levels of ChREBPα were examined by immunoblotting with anti‐ChREBPα. Figure S4: RNF8 accelerates the degradation rate of ChREBPα in mouse hepatocytes. Cells were first transduced with Ad‐ChREBPα with or without Ad‐RNF8 before treatment with Cycloheximide (CHX 100 μg/mL) for the indicated times. The relative expression of ChREBPα protein was normalized to the loading control GAPDH. The ChREBPα level at 0 h time point represents 100%. Data are presented as mean ± S.D. from three independent experiments. Figure S5: Effects of C‐terminal deletion of ChREBPα protein on response to RNF8‐mediated degradation; 293 Ad cells were first transfected with expression vectors encoding either Flag‐Chrebpα full‐length, 1‐200aa, or 1‐400aa truncation mutants prior to treatment with Ad‐Rnf8. The cells were then harvested for immunoblotting [file FSB2-40-e71830-s001.pdf]

SI Figures & Figure legends

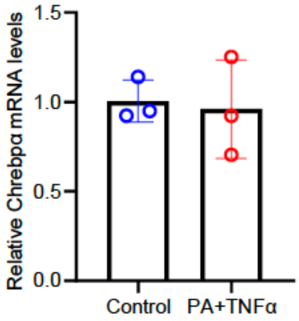

Figure S1. Combined treatment with Palmitate and TNFα does not affect Chrebpα mRNA levels in primary mouse hepatocytes. Cells were treated with Palmitate (300 μM, 24 hrs) and TNFα (10 ng/mL, 6 hrs) before being harvested for RNA extraction and mRNA analysis by QPCR. Data are presented as mean ± SD.

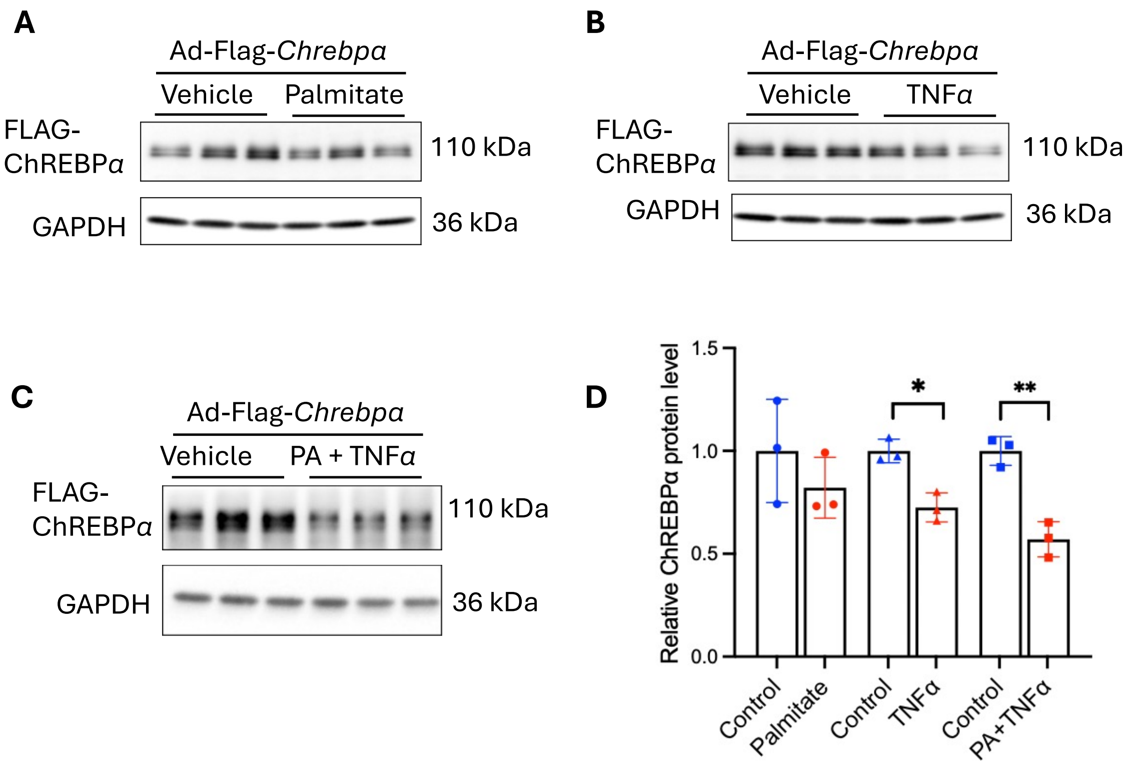

Figure S2. Immunoblot analysis of ChREBPα expression in PMHs transduced with Ad-Flag-*ChREBPα* and then treated with palmitate (300 μM, 24 hrs) alone (**A**), TNFα (10 ng/mL, 6 hrs) alone (**B**), or palmitate (300 μM, 24 hrs) plus TNFα (10 ng/mL, 6 hrs) (**C**). ChREBPα protein levels were quantified by *Image J* (**D**). The data were plotted as mean ± SD. * p-value < 0.05, ** p-value < 0.01 by the student's t-test.

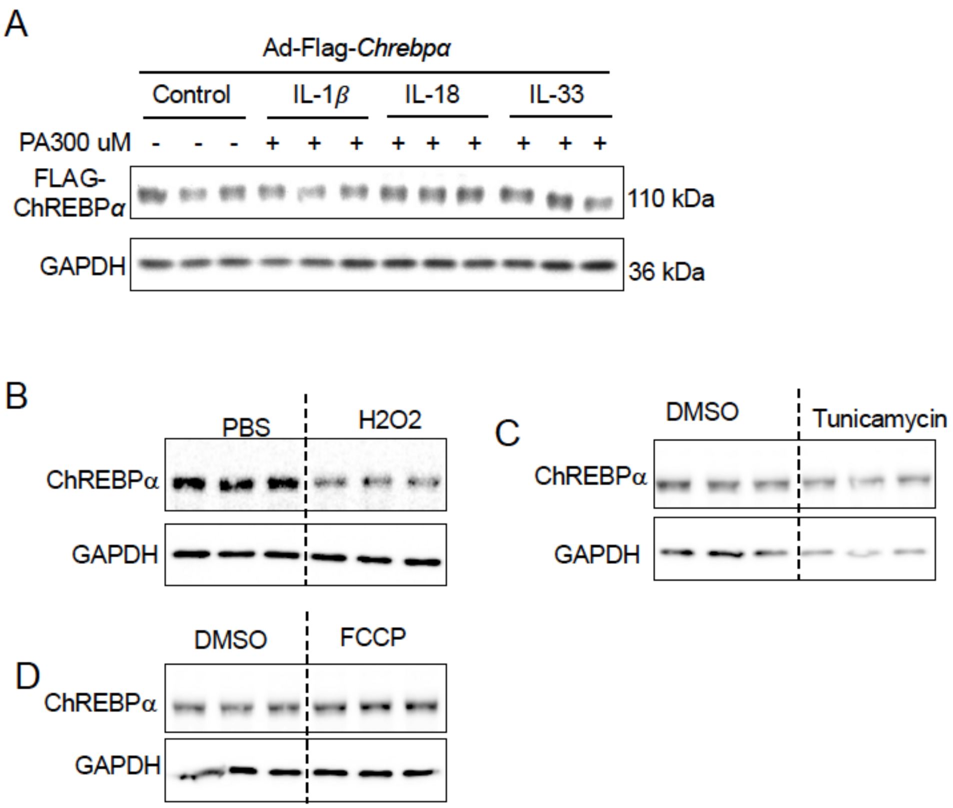

Figure S3. (A) Effects of common pro-inflammatory cytokines on ChREBPα protein in hepatocytes. PMHs were transduced with *Ad-Chrebp*α and treated with palmitate (300 μM) and a cytokine—either IL-1β (15 ng/mL), IL-18 (5 ng/mL), or IL-33 (50 ng/mL). **(B-D)** Effects of MASH-associated stress signals on ChREBPα protein levels in hepatocytes. PMHs were transduced with *Ad-Chrebp*α and treated with H_2O_2 (400 μM), tunicamycin (2 μM), and FCCP (2 μM) for 6 hours before harvest. The protein levels of ChREBPα were examined by immunoblotting with anti-ChREBPα.

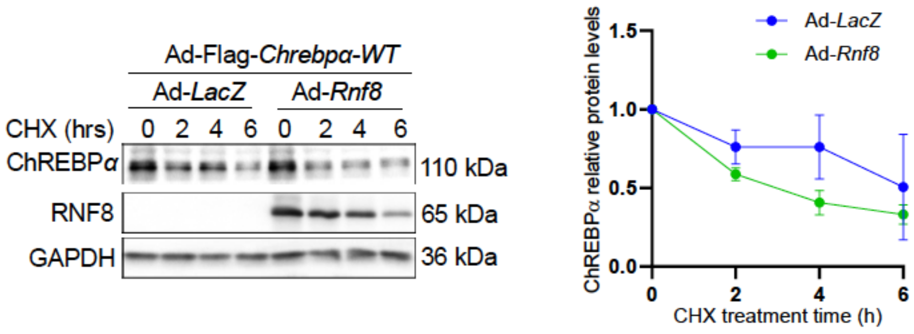

Figure S4. RNF8 accelerates the degradation rate of ChREBPα in mouse hepatocytes. Cells were first transduced with Ad-ChREBPα with or without Ad-RNF8 before treatment with Cycloheximide (CHX 100 μg/ml) for the indicated times. The relative expression of ChREBPα protein was normalized to the loading control GAPDH. The ChREBPα level at 0 h time point represents 100%. Data are presented as mean ± S.D. from three independent experiments.

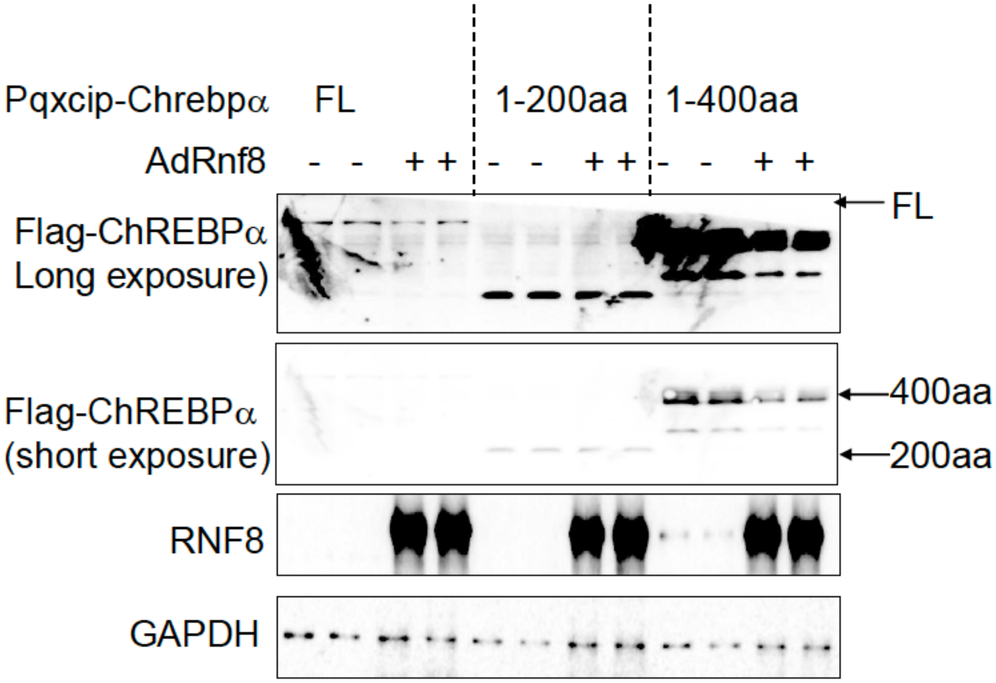

Figure S5. Effects of C-terminal deletion of ChREBPα protein on response to RNF8-mediated degradation. 293AD cells were first transfected with expression vectors encoding either Flag-Chrebpα full-length, 1-200aa, or 1-400aa truncation mutants prior to treatment with Ad-*Rnf8*. The cells were then harvested for immunoblotting to examine the abundance of ChREBPα protein using anti-Flag.
